# The Effect of Cold Periods on the Biological Cycle of *Marchalina hellenica*

**DOI:** 10.3390/insects13040375

**Published:** 2022-04-11

**Authors:** Spiros D. Dafnis, Sofia Gounari, Chris E. Zotos, George K. Papadopoulos

**Affiliations:** 1 Department of Crop Science, Agricultural University of Athens, Iera Odos 75, 11855 Athens, Greece; czotos@aua.gr (C.E.Z.); gpapadop@aua.gr (G.K.P.); 2 Laboratory of Apiculture, Institute of Mediterranean Forest Ecosystems, Hellenic Agricultural Organization, DEMETER, Terma Almanos, 14180 Athens, Greece; sgounari@fria.gr

**Keywords:** runs, cumulative logit model, forecasting model, non-wood forest products, climate change, honeydew flow

## Abstract

**Simple Summary:**

The most important honeydew-producing insect in Greece is *Marchalina hellenica* (Coccoidea: Marchalinidae), which is a parasite on pine trees. The current work is part of an ongoing research project aiming to provide knowledge on honeydew-producing insects and the impact of critical factors (climate, beekeeping manipulations, honeydew-producing insect phenology) on honeydew honey production. Empirical evidence indicates that among the weather factors, the most important one, at least for spring honeydew secretions, appears to be temperature and, more specifically, the existence of cold winter days. Presently, we investigate the effect of cold periods in February on the life cycle of *Marchalina hellenica*. Our primary goal is to help beekeepers plan the timely exploitation of honeydew secretions of pine trees. Such a potential will be beneficial for beekeepers, the rural economy, and forest protection. It should be noted that current results highlight the impact of climate change in the field of entomology, and they indicate that the life cycle of *Marchalina hellenica* is expected to be drastically shorter.

**Abstract:**

Climate change is considered a major factor affecting honeybees’ behavior and productivity with major consequences in both honey and agricultural production. Many research studies have expressed serious concerns about the mass losses of bee colonies and the role of bees as pollinators, while others have underlined important issues for the impact of the increase in temperature on honeybee abundance and honey yields. In the present work, we draw our attention to *Marchalina hellenica*, which is the most important honeydew-producing insect in Greece. A statistically significant forecasting model for the effect of cold periods in February on the life cycle of the insect is constructed, with the aid of the Cumulative Logit Model and the theory of runs. The forecasting model may help beekeepers plan the timely exploitation of honeydew secretions of pine trees, which will be beneficial for beekeepers, the rural economy, and forest protection. The new suggested model also indicates that, in view of the climate change scenarios seen in the literature, the life cycle of *M. hellenica* is expected to be drastically shorter.

## 1. Introduction

Climate change is considered a major factor affecting honeybees’ behavior and productivity with major consequences in both honey and agricultural production [1]. Even though the main role of beekeeping is honey production [2,3], the maintenance of honeybees contributes significantly to the pollination activity. It should be noted that honeybees, as pollinators, provide valuable services to agricultural [4,5] and natural ecosystems with significant impacts on biodiversity and food security [6]. Therefore, many research studies have expressed serious concerns about the mass losses of bee colonies and the role of bees as pollinators [7,8], while others have underlined important issues for the impact of climate change on honeybee abundance and honey yields (see e.g., [9]). The impact of the changing climate is anticipated to be serious in the region of the East Mediterranean and the Middle East, as positive warming trends and increasing aridity have been identified by many research studies [10,11]. Small islands are even more vulnerable regarding agricultural production and trade under the current climate change scenarios [12].

The impact of weather is critical in the availability of pollen and nectar. Weather also influences honeydew-producing insects as honeydew is significant for honey production. The honeydew producers, mainly Coccoidea or Aphidoidea, are insects with highly modified mouthparts and digestive systems [13]. They produce droplets of honeydew as they feed from the phloem sap of forest plant species. These droplets, rich in carbohydrates, turn into honeydew honey by the honeybees.

In Europe, 50% of the annual honey production is honeydew honey. In Greece, the respective percentage is 70%. The honeydew honey produced in Greece is mainly pine, fir, and oak honey. Pine honeydew honey is an economically important non-wood forest product (NWFP) from eastern Mediterranean *Pinus* spp. forests. It is of high nutritional value, rich in antioxidants and trace elements (calcium, magnesium, iron, zinc) with high antiseptic action and therapeutic properties [14]. It is only produced in Turkey and Greece. While the annual honey production of Greece is estimated at 13,000–17,000 tons, pine honey constitutes about 60% of the total annual honey production [15].

The most important honeydew-producing insect in Greece is *Marchalina hellenica* (Coccoidea: Marchalinidae), which is a parasite on pine trees. It has a generation per year. It overwinters as third-instar nymphs and produces large quantities of honeydew from March to April, before its last molt to a female adult. The knowledge of the biological cycle of *Marchalina hellenica (M. hellenica)* is highly important to beekeepers, as it affects the transportation of the beehives, the rate of honeydew production, and the amount of honey produced [9,16,17]. It should also be noted that the maintenance of the economic activity also protects the forest [18]. Although, in Greece, the phenology of *M. hellenica* has been studied sufficiently [19,20], this is not the case regarding the relationship between the biological cycle of the insect (which directly affects the amount of honey secretion) and the weather conditions.

Empirical evidence indicates that among the weather factors, the most important one, at least for spring honeydew secretions, appears to be temperature and, more specifically, the existence of cold winter days. This important empirical knowledge has been recently proven by Gounari et al. [16]. In the latter work, the authors employed the Cumulative Logit Model to prove that temperature, and more specifically, the number of cold days in February, is a significant parameter to the life cycle of *M. hellenica*, and they constructed a statistically significant forecasting model. It should be noted that the findings of that work coincide with knowledge also derived by other species, which are not able to finish the developmental cycle or to continue feeding in spring without the existence of a sufficient number of low-temperature days [21].

In the present paper, the forecasting model is enriched. The theory of runs (see [22]) is employed along with the Cumulative Logit Model, and it is proved that the number of cold periods in February is also a significant parameter to the life cycle of *M. hellenica*. It should be noted that, as far as the authors know, this is the first time in academic research that the theory of runs has been combined with ordinal regression models. However, the theory of runs has been, independently, applied to several agricultural problems (see, e.g., [23]).

The forecasting model currently developed may stress the effect of climate change and the absence of cold periods and help beekeepers plan the timely exploitation of honeydew secretions of pine trees, which will be beneficial for beekeepers, the rural economy, and forest protection.

## 2. Materials and Methods

*M. hellenica* is a normal non-cyst-forming coccid, with the life cycle of the females having three immature stages or instars and that of the males having four immature stages [19]. A detailed description of the life cycle of *M. hellenica* can be found in [19].

**Figure 1:**
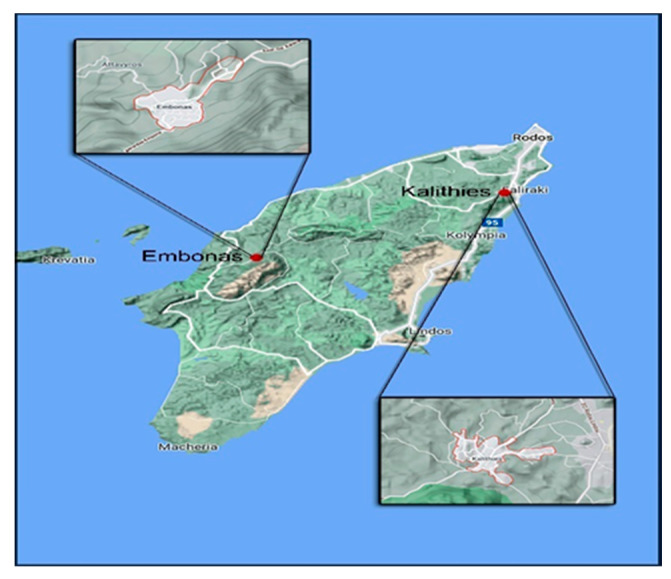
Map of Rhodes with the areas of interest.

Regarding the study area, Rhodes is the largest Greek island in the Dodecanese and is located in the south-eastern part of the Aegean Sea. The main honey types produced are pine and thyme honey. Pine forests cover most of the island, reaching, in some places, up to the sea. Kalithies is at the north-eastern part of the island, while Embonas is at the center-west (Figure 1).

We used the data collected by Gounari [20] regarding the biological cycle of the insect in the two areas of interest (for a detailed presentation of sampling procedure and data collection, see [20]). We should reiterate that temperature constitutes a parameter of major importance when observing the life cycle of *M. hellenica*. Therefore, the winter temperature in the areas of interest, Kalithies and Embonas, during the period from 2014 to 2019 was monitored. The climatic variable of temperature for the two regions was recorded in two different weather stations of IERSD/NOA (Institute for Environmental Research, National Observatory of Athens). More specifically, interest was focused on the minimum daily winter (December to February) temperature. The basic descriptive statistics of temperature are presented in Table 1.

Next, the process of completion of adult insects was observed using the available data from 2014 to 2019 for the island of Rhodes. The fortnight of integration as well as other characteristics are presented in Table 2. More specifically, the fortnight of adult *M. hellenica* was categorized in J=5 categories. The first category (1) corresponds to the occasion when *M. hellenica* becomes an adult during the second fortnight of March, category (2) when the completion takes place during the first fortnight of April, category (3) when the completion takes place during the second fortnight of April, category (4) when the completion takes place during the first fortnight of May, and category (5) when the completion takes place during the second fortnight of May.

Due to some differences between the two regions regarding the time when *M. hellenica* completes its biological cycle, an independent samples t-test to check whether this difference was statistically significant was performed. The results showed that there was no statistical difference (*p* > 0.050) in the fortnight of completion between the two regions, indicating that the data should not be differentiated per region for the purposes of this analysis.

### 2.1. Statistical Analysis

During recent decades, a wide range of problems in several research areas such as Agriculture, Biology, Finance, Meteorology, Quality Control, and Systems’ Reliability (see e.g., [22,23]) has been modeled by classifying the experimental trials in two exclusive categories, considering sequences of binary trials (with values 0 or 1) and studying the sequences of outcomes. This study usually involves searching for the greatest concentration of outcomes of a specific type. Such a concentration is traditionally measured by the enumeration of runs of (*k* ≥ 1) 1s. In the present work, an ordinal regression model employing runs was proposed. Depending on the special features of the specific application under study, several ways for counting runs have been proposed in the literature. Currently, we should draw attention to the Type I counting scheme [22,24]. According to the Type I counting scheme, a run of length *k* is registered when *k* consecutive 1 s are observed, and the (*k* + 1)th consecutive 1 is considered as the first 1 of the next run.

**Figure 2:**
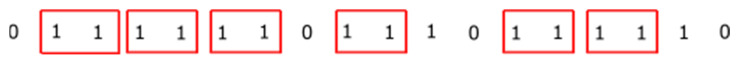
Runs of length *k* = 2.

**Figure 3:**
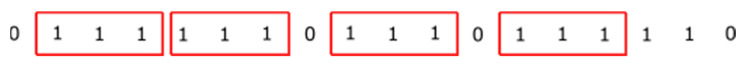
Runs of length *k* = 3.

**Figure 4:**
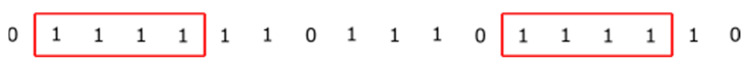
Runs of length *k* = 4.

As an example of the Type I counting scheme for runs of length k, let us assume that by examining 18 consecutive days, the sequence 011111101110111110 of days that have been classified as cold or not-cold ones (1 stands for a cold day and 0 stands for a not-cold day according to prespecified criteria) has been observed. Then, we may count, for example, six different runs of length k=2, four different runs of length k=3, or two different runs of length k=4 (see Figure 2, Figure 3 and Figure 4).

In the present work, the theory of runs was employed along with an ordinal regression model, and the number of cold periods in February is a significant parameter to the life cycle of *M. hellenica*.

Ordinal regression models are used when the response variable is ordinal with more than two categories. The present work used the Proportional Odds Model (POM), which assumes that the relationship between predictor and response variables is independent of the response variable’s category [25].

Let Y be a response variable with J categorical outcomes, denoted by 1,2,…,J, and let x be a vector of covariates (independent/predictor variables). Let $\pi_{1}\left(x\right)$, $\pi_{2}\left(x\right)$,…, $\pi_{J}\left(x\right)$ also be the probabilities of the J-ordered categories of the response variable when covariates have the value x. Then, the Proportional Odds Model is defined as follows:

(1) $$\gamma_{j}=Pr\left(Y\leq j|x\right)=\frac{1}{1+e^{-\left(a_{j}+\beta^{\prime }x\right)}},j=1,\;2,\ldots ,J-1$$

where $a_{j}$ is the intercept in category *j* and β is a vector of regression coefficients corresponding to x (we denote by $\beta^{\prime }$ the transpose of the matrix β).

In logit form, the Proportional Odds Model transforms as follows:

(2) $$\mathrm{logit}\left(\gamma_{j}\right)=\ln\left(\frac{\gamma_{j}}{1-\gamma_{j}}\right)=\ln\left(\frac{Pr(Y\leq j|x)}{Pr(Y>j|x)}\right)=a_{j}+\beta^{\prime }x,j=1,\;2,\ldots ,J-1$$

The $a_{j}$ are increasing in *j*, as Pr(Y≤j|x) increases in *j* for fixed x, and the logit is an increasing function of this probability [26].

It must be noted that the POM utilizes the cumulative probabilities:

$$\gamma_{j}=Pr\left(Y\leq j|x\right)={\;\pi }_{1}\left(x\right)+{\;\pi }_{2}\left(x\right)+\ldots +{\;\pi }_{j}\left(x\right)$$

and the regression coefficients vector β does not depend on j, meaning that the relationship between Y and x is independent of j.

In addition, the cumulative odds ratio

$$OR=\frac{Pr\left(Y\leq j|x_{1}\right)/Pr\left(Y>j|x_{1}\right)}{Pr\left(Y\leq j|x_{2}\right)/Pr\left(Y>j|x_{2}\right)}\;\;=e^{\beta^{\prime }\left(x_{1}-x_{2}\right)},$$

is independent of *j* and depends only on the difference between the covariate values, $x_{1}-x_{2}$ [26].

In the present work, the vector of independent variables ***x*** consists of a single variable, $X_{k}$, which is obtained through the theory of runs of length k, for different values of the parameter k. As a result, the regression coefficients vector β consists of a single coefficient, $\beta_{1}$, as well. Based on these, the model becomes,

(3) $$\gamma_{j}=Pr\left(Y\leq j|X_{k}\right)=\frac{1}{1+e^{-\left(a_{j}+\beta_{1}X_{k}\right)}},j=1,\;2,\ldots ,J-1$$

and in logit form,

(4) $$\mathrm{logit}\left(\gamma_{j}\right)=\ln\left(\frac{\gamma_{j}}{1-\gamma_{j}}\right)=\ln\left(\frac{Pr\left(Y\leq j|X_{k}\right)}{Pr\left(Y>j|X_{k}\right)}\right)=a_{j}+\beta_{1}X_{k},j=1,\;2,\ldots ,J-1$$

#### Determining the Variables

Gounari et al. [16] employed an ordinal regression model to predict the fortnight of completion of the biological cycle of the honeydew-producing insect, *M. hellenica*, based on the number of cold days in February. Τhe threshold for classifying a day of February as a cold one was set to the 20% percentage $\left(P_{20}\right)$ of the dataset of the minimum daily winter temperature, which was calculated to be equal to 7.3 °C (see Table 1), i.e., any day with a minimum temperature less than 7.3 °C was classified as a cold day. The current study also considered another statistically significant criterion, the number of cold periods in February. In order for the definition of a cold period to be possible, a different classification must be made. Any day in February is classified as a relatively cold day (rcd) if the daily minimum temperature is lower than the 30% percentage $\left(P_{30}\right)$ of the dataset of the minimum daily winter temperatures, which, in this case, is 8.5 °C (see Table 1). Therefore, any day in February with a minimum temperature less than 8.5 °C is classified as a rcd. It should be noted that there is strong empirical evidence that the number of days in February when the minimum daily temperature is lower than a specific threshold has an analogous effect on the prolongation of the completion of the biological cycle of *M. hellenica,* and both temperature thresholds of 7.3 °C and 8.5 °C coincide with the practitioners’ experience. A run of k consecutive rcds is referred to as a cold period. It is proven that the number of cold periods in February is a significant parameter to the life cycle of *M. hellenica,* a predictability that is of vital importance to beekeepers. It should be noted that the criterion of the number of cold days, employed by Gounari et al. [16], is now replaced by the number of runs of consecutive rcds (cold periods). The daily temperature threshold is now relaxed as a run has a cumulative effect. The new results of this work regarding the effect of the number of k (k=2,3) consecutive runs of rcds in February on the life cycle of *M. hellenica* is now presented. Table 3 describes in detail the dependent variable (Y) and the respective independent variable $\left(X_{k}\right)$ that was used in the one-dimensional ordinal regression model for the values k=2,3. Moreover, Table 4 gives the realizations of ${Χ}_{2}$ and ${Χ}_{3}$ in the experimental data every year from 2014 to 2019.

## 3. Results

In this section, the results regarding the three different statistically significant criteria to the life cycle of *M. hellenica* (number of cold days in February, number of runs of two consecutive rcds in February, number of runs of three consecutive rcds in February) are presented. Before the presentation of the new results regarding the criteria of runs of rcds (cold periods), the results of Gounari et al. [16] regarding the effect of the number of cold days in February (Criterion 1) are presented.

### 3.1. Criterion 1: Number of Cold Days in February

As it is already mentioned in the Introduction, Gounari et al. [16] proved the effect of temperature, and more specifically, the number of cold days in February, to the life cycle of *M. hellenica.* They employed the POM to prove that for each additional cold day in February, the Odds Ratio (OR) of *M. hellenica* to complete its cycle in a specific fortnight decreased by 52.15%. We now proceed to the new results regarding the criteria of cold periods in February.

### 3.2. Criterion 2: Number of Runs of Two Consecutive Rcds in February

The POM was employed with the dependent variable Y and the independent variable $X_{2}$. The assumption that all the logit surfaces, lines in this case, are parallel (i.e., proportional odds assumption) was tested and the null hypothesis of parallelism of the logit lines with respect to x was accepted (*p* > 0.050). The POM was tested, and it was statistically significant (p<0.050) (Table 5). The odds ratio estimate, OR = 0.363, indicated that the odds of *M. hellenica* to complete its cycle in a specific fortnight were multiplied by 0.363 for each additional run of two consecutive rcds in February. In other words, for each additional run of two consecutive rcds in February, the odds of *M. hellenica* to complete its cycle in a specific fortnight decreased by 63.7%. Therefore, the number of two consecutive rcds was a more critical factor to the completion time of the insect’s biological cycle than the number of cold days.

**Figure 5:**
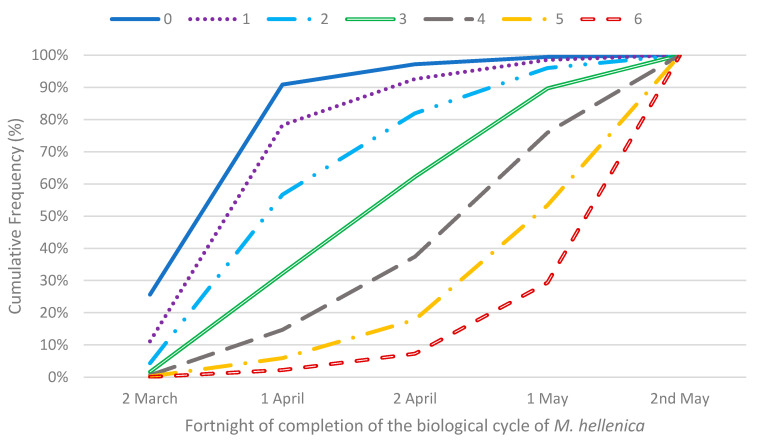
Plots of cumulative frequencies for fortnight of completion of the biological cycle of M. hellenica and *k* = 2.

A diagram of the observed cumulative frequencies for the fortnight of completion of the biological cycle of *M. hellenica* (Figure 5) is also given. Each curve of the plot corresponds to the number of runs of rcds, from zero to six, which was recorded for the month of February. The cumulative probability curves on the left side of the plots correspond to a lower number of runs of rcds. More specifically, these curves express the low probability that *M. hellenica* will become an adult in one of the subsequent reference fortnights. Figure 5 reveals that as the number of runs of two consecutive rcds increased, the probability of completion of the biological cycle of *M. hellenica* in a following fortnight also increased. Considering Equation (2) and the estimated coefficients in Table 5, the cumulative probabilities of adult *M. hellenica* biological cycle completion and, consequently, the probability of every specific fortnight were computed (Table 6).

### 3.3. Criterion 3: Number of Runs of Three Consecutive Rcds in February

The POM was employed with the dependent variable Y and the independent variable $X_{3}$. The assumption of parallelism was successfully tested (p>0.050). The POM was proved to be statistically significant (p<0.050) (Table 7). According to Table 7, the addition of a run of three (consecutive) rcds in February decreased the OR of *M. hellenica* to complete its biological cycle within a specific fortnight by 79%. In comparison with the previous case, the increase in the run’s length per rcd led to a further decrease in the corresponding OR by 15.3%.

**Figure 6:**
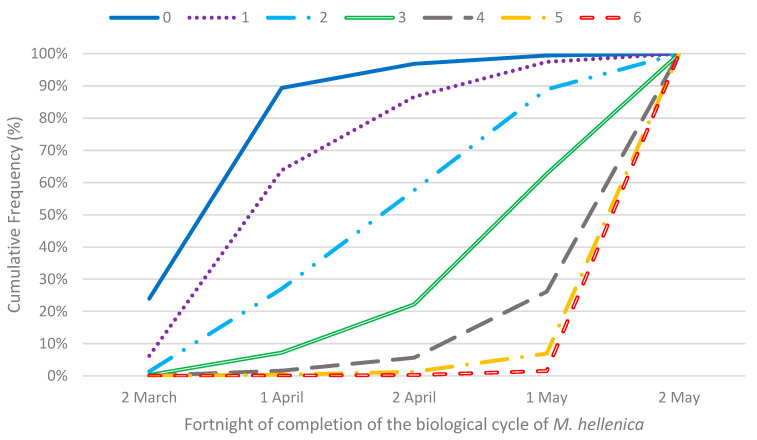
Plots of cumulative frequencies for fortnight of completion of the biological cycle of M. hellenica and *k* = 3.

Figure 6 and Table 8 are now given. In Figure 6, the cumulative probability curves corresponding to six runs of three rcds indicated that the probability of completion of the biological cycle of *M. hellenica* in one of the initial reference fortnights was almost zero.

Let $\mu_{i}$ denote the average minimum temperature in day i (i=1,…,28) of February for the area of Embonas of Rhodes Island and all years from 2014 to 2019, and $W_{k}$ denote the number of runs of k consecutive values of $\mu_{i}$ less than 8.5 °C. We may calculate that $W_{2}=7$ and $W_{3}=4$. In the Discussion, these values are compared to the respective values under climate change scenarios and the impact of climate change is highlighted.

## 4. Discussion

Current work is part of an ongoing research project aiming to provide additional knowledge on honeydew-producing insects and the impact of critical factors (climate, beekeeping manipulations, honeydew-producing insect phenology) on honeydew honey production. Its primary goal is to help beekeepers plan the timely exploitation of honeydew secretions of pine trees. Such a potential results in several personal and social benefits. More specifically, the timely exploitation of honeydew secretions of pine trees:

- Reduces production costs of honey with the simultaneous increase in competitiveness, for a large part of beekeepers, who exploit honeydew insect secretions to collect honey, through streamlining the transportation of beehive colonies.
- Reduces transportation costs by reducing beekeepers’ transfers for beehives’ transport and on-site inspections. It also reduces the stress of vehicles and the likelihood of an accident on the road.
- Reduces labor costs due to transportation reduction and on-site inspections.
- Reduces bee colony losses. Part of the population of bees are lost and/or dying during transport. Frequent inspections and inappropriate harvesting can cause looting, which may result in the loss of entire flocks or in the killing of Queens.
- Increases production per hive. The choice of region and the timely transfer and removal of bees from one honeydew increases the overall performance of honeydew production.

Presently, the recent work of Gounari et al. [16] is expanded by the addition of extra criteria, which include the number of cold periods in February (number of runs of rcds of length k=2 and k=3). The new results of this work were obtained by the combination of ordinal regression and the theory of runs. It should also be noted that, as far as the authors know, this is the first time in academic research that the theory of runs has been combined with ordinal regression models. The authors strongly believe that such a combination can be profitably applied to other scientific fields as well, such as biomedical engineering (see [27]).

The impact of temperature on the biological cycle of *M. hellenica* can be readily seen in Table 7 and Table 8. For instance, in the absence of runs of two consecutive rcds in February or three consecutive rcds in February, *M. hellenica* will have completed its biological cycle by April’s second fortnight with a probability 97.17% and 96.86%, respectively (employing Criterion 1, the respected probability was calculated to be 98.35%, see [16]). When February has four runs of two consecutive rcds, the probability that *M. hellenica* will have completed its biological cycle by April’s second fortnight decreases by about 60% compared to the case when there are no consecutive rcds. On the other hand, if February has four runs of three consecutive rcds, the respective probability now decreases by about 91%. It is also worth noting that when February has two runs of two consecutive rcds, the most probable fortnight of *M. hellenica*’s biological cycle’s completion is the first fortnight of April with a probability of around 52%. When February has two runs of three consecutive rcds, the most probable fortnight of *M. hellenica*’s biological cycle’s completion is the second fortnight of April with a probability of about 31%.

Therefore, it may be seen that the combination of runs and ordinal regression highlighted and further quantified the effect of temperature to the life cycle of *M. hellenica.* Moreover, the new theoretical tools enable an estimation of the effect of climate change to the field.

The atmospheric temperature is projected to continue to rise throughout the 21st century in most areas of the planet (see [28] and the references therein). In particular, on the basis of average results over a number of climate model simulations, the average atmospheric temperature is expected to increase by 1.8–4 °C by 2100, depending on developments in the concentration of greenhouse gas emissions [28]. The increase in temperature is expected to be greater at higher latitudes and in continental regions as opposed to the oceans. Therefore, let us consider the following three climate change scenarios: Scenario (i) the average minimum atmospheric temperature is expected to increase by 1.8 °C (optimistic scenario); (ii) the average minimum atmospheric temperature is expected to increase by 2.9 °C (moderate scenario); (iii) the average minimum atmospheric temperature is expected to increase by 4 °C (pessimistic scenario). Let $\mu_{d}^{\left(s\right)}\;$denote the expected minimum temperature in day d (d=1,…,28) of February in the year 2100 according to the climate change scenario s (s=1,2,3), and $W_{k}^{\left(s\right)}$ denote the expected number of runs of k consecutive values of $\mu_{d}^{\left(s\right)}$ less than 8.5 °C, according to the climate change scenario s (s=1,2,3). It holds true that $\mu_{d}^{\left(1\right)}=\mu_{d}+1.8\;{}^{\circ}C,\;\mu_{d}^{\left(2\right)}=\mu_{d}+2.9\;{}^{\circ}C,\;\mu_{d}^{\left(3\right)}=\mu_{d}+4\;{}^{\circ}C.$ Therefore, the values of $W_{k}^{\left(s\right)}$ for k=2,3 may now be calculated, which are all equal to zero. It may be concluded that the value of $W_{2}$ has decreased by seven units and the value of $W_{3}$ has decreased by 4, even by assuming the optimistic climate change scenario. In view of the previous results in this section, this drastic decrease in the number of the expected runs indicates that, even in the optimistic scenario, the life cycle of *M. hellenica* will be significantly shortened. We recall that, according to our model, if, for example, the number of runs of two consecutive rcds reduces by one, the odds of *M. hellenica* to complete its cycle in a specific fortnight are multiplied by 1/0.363, or they increase by 175%.

Therefore, the new suggested model enables the incorporation of the results in the climate change scenarios seen in the literature. It is currently shown that the life cycle of *M. hellenica* is expected to be drastically shorter, which will affect the forests, the rural communities, and society in general.

## Figures and Tables

**Table 1:** Descriptive statistics for minimum daily winter temperatures of Rhodes island.

Descriptive Statistics	Average	Min	Max	s.d. ^1^	$P_{20}{\;}^{2}$	$P_{25}{\;}^{3}$	$P_{30}{\;}^{4}$	$P_{50}{\;}^{5}$
Kalithies	11.51	0.70	18.90	2.85	9.60	10.20	10.70	12.00
Embonas	8.06	−1.00	17.60	3.10	5.42	6.40	7.00	8.60
Total	9.95	−1.00	18.90	3.43	7.30	7.90	8.50	10.50

^1^ s.d.: standard deviation, ^2^ $P_{20}$: 20th percentile, ^3^ $P_{25}$: 25th percentile, ^4^ $P_{30}$: 25th percentile, ^5^ $P_{50}$: median, temperature is measured in degrees Celsius (°C).

**Table 2:** Descriptive statistics for the fortnight of completion of adult insects.

Fortnight Category	Codes	Number of Cases	Marginal Percent (%)	Cumulative Percent (%)
2nd fortnight of March	1	1	9.10	9.10
1st fortnight of April	2	4	36.30	45.40
2nd fortnight of April	3	2	18.20	63.60
1st fortnight of May	4	2	18.20	81.80
2nd fortnight of May	5	2	18.20	100.00
Valid		11	100.00	
Missing		1		
Total		12		

**Table 3:** Description of variables of ordinal regression model.

Variables	Symbol	Description	Unit/Scale of Measurement
Dependent variable			
Fortnight	Y	Fortnight of completion of the biological cycle of M. hellenica	Ordinal variable is measured in five categories
Independent variable			
Number of runs (k=2)	${Χ}_{2}$	Number of two	Ratio scale variable
		consecutive	
		rcds during February	
Number of runs (k=3)	${Χ}_{3}$	Number of three	Ratio scale variable
		consecutive	
		rcds during February	

**Table 4:** Runs occurrences in the experimental data.

	February	${Χ}_{2}$	${Χ}_{3}$	Y
Kalithies	2014	0	0	2
	2015	3	4	3
	2016	1	1	4
	2017	1	2	2
	2018	0	0	1
	2019	0	0	2
Embonas	2014	−	−	4
	2015	7	9	5
	2016	3	4	4
	2017	4	7	5
	2018	1	4	2
	2019	5	7	3

**Table 5:** Statistical results of POM for k=2.

	Parameters	Estimate	Std. Error	Wald	D.F.	*p*-Value	
Threshold							
$a_{1}$	[1]	−1.065	1.160	0.843	1	0.359	
$a_{2}$	[2]	2.295	1.318	3.031	1	0.082	
$a_{3}$	[3]	3.536	1.521	5.406	1	0.020	
$a_{4}$	[4]	5.200	1.952	7.099	1	0.008	
Location		$\beta_{1}$					$Exp\left(\beta_{1}\right)$
$X_{2}$		−1.013	0.432	5.512	1	0.019	0.363

**Table 6:** Prediction of the fortnight of *M. hellenica’s* biological cycle completion based on the number of runs of two consecutive rcds in February (*k* = 2).

Number of Runs of Two Consecutive Rcds in February	Fortnight	Cumulative Distribution Function (CDF)	Probability Mass Function (PMF)	Cumulative Odds (CO)
0	2 March	0.2564	0.2564	0.3447
	1 April	0.9085	0.6521	9.9244
	2 April	0.9717	0.0632	34.3293
	1 May	0.9945	0.0228	181.2722
	2 May	1	0.0055	
1	2 March	0.1113	0.1113	0.1252
	1 April	0.7828	0.6715	3.6038
	2 April	0.9257	0.1429	12.4659
	1 May	0.9850	0.0593	65.8250
	2 May	1	0.0150	
2	2 March	0.0435	0.0435	0.0455
	1 April	0.5668	0.5234	1.3087
	2 April	0.8191	0.2522	4.5267
	1 May	0.9598	0.1408	23.9029
	2 May	1	0.0402	
3	2 March	0.0162	0.0162	0.0165
	1 April	0.3221	0.3059	0.4752
	2 April	0.6218	0.2996	1.6438
	1 May	0.8967	0.2749	8.6798
	2 May	1	0.1033	.
4	2 March	0.0060.	0.0060	0.0060
	1 April	0.1472	0.1412	0.1726.
	2 April	0.3738	0.2266	0.5969
	1 May	0.7591	0.3854	3.1519
	2 May	1	0.2409	
5	2 March	0.0022	0.0022	0.0022
	1 April	0.0590	0.0568	0.0627
	2 April	0.1781	0.1192	0.2168
	1 May	0.5337	0.3556	1.1445
	2 May	1	0.4663	
6	2 March	0.0008	0.0008.	0.0008
	1 April	0.0222	0.0215	0.0228
	2 April	0.0730	0.0507	0.0787
	1 May	0.2936	0.2206	0.4156
	2 May	1	0.7064	

Note: The column of CDF indicates the probability of the biological cycle completion of *M. hellenica* lower or equal to a specific fortnight *j* ($\gamma_{j}$). The column PMF indicates the probability of the biological cycle completion of *M. hellenica* equal to a specific fortnight *j* and can be calculated as: $\gamma_{j}-\gamma_{j-1}$.

**Table 7:** Statistical results of POM for k=3.

Threshold		Estimate	Std. Error	Wald	D.F.	*p*-Value	
$a_{1}$	Fortnight = [1]	−1.153	1.145	1.016	1	0.314	
$a_{2}$	Fortnight = [2]	2.128	1.236	2.964	1	0.085	
$a_{3}$	Fortnight = [3]	3.428	1.489	5.298	1	0.021	
$a_{4}$	Fortnight = [4]	5.205	2.039	6.517	1	0.011	
Location		$\beta_{1}$					$Exp\left(\beta_{1}\right)$
$X_{3}$		−1.561	0.621	6.230	1	0.012	0.210

**Table 8:** Prediction of the fortnight of *M. hellenica’s* biological cycle completion based on the number of runs of three consecutive rcds in February (k=3).

Number of Runs of Three Consecutive Rcds in February	Fortnight	Cumulative Distribution Function (CDF)	Probability Mass Function (PMF)	Cumulative Odds (CO)
0	2 March	0.2399	0.2399	0.3157
	1 April	0.8936	0.6537	8.3981
	2 April	0.9686	0.0750	30.8150
	1 May	0.9945	0.0260	182.1809
1	2 May	1	0.0055	
	2 March	0.0622	0.0622	0.0663
	1 April	0.6381	0.5759	1.7630
	2 April	0.8661	0.2280	6.4689
	1 May	0.9745	0.1084	38.2445
	2 May	1	0.0255	
2	2 March	0.0137	0.0137	0.0139
	1 April	0.2701	0.2564	0.3701
	2 April	0.5759	0.3058	1.3580
	1 May	0.8892	0.3133	8.0285
	2 May	1	0.1108	
3	2 March	0.0029	0.0029	0.0029
	1 April	0.0721	0.0692	0.0777
	2 April	0.2218	0.1497	0.2851
	1 May	0.6276	0.4058	1.6854
	2 May	1	0.3724	
4	2 March	0.0006	0.0006	0.0006
	1 April	0.0160	0.0154	0.0163
	2 April	0.0565	0.0404	0.0598
	1 May	0.2613	0.2049	0.3538
	2 May	1	0.7387	
5	2 March	0.0001	0.0001	0.0001
	1 April	0.0034	0.0033	0.0034
	2 April	0.0124	0.0090	0.0126
	1 May	0.0691	0.0567	0.0743
	2 May	1	0.9309	
6	2 March	0.0000	0.0000	0.0000
	1 April	0.0007	0.0007	0.0007
	2 April	0.0026	0.0019	0.0026
	1 May	0.0154	0.0127	0.0156
	2 May	1	0.9846	

Note: The column of CDF indicates the probability of the biological cycle completion of *M. hellenica* lower or equal to a specific fortnight *j* ($\gamma_{j}$). The column PMF indicates the probability of the biological cycle completion of *M. hellenica* equal to a specific fortnight *j* and can be calculated as: $\gamma_{j}-\gamma_{j-1}$.

## Data Availability

Data are available on request.
